# Computational Intelligence in Biomedical Science and Engineering

**DOI:** 10.1155/2012/160356

**Published:** 2012-12-03

**Authors:** Yen-Wei Chen, Ikuko Nishikawa, Shinichi Tamura, Bao-Liang Lu, Huiyan Jiang

**Affiliations:** ^1^College of Information Science and Engineering, Ritsumeikan University, Shiga 525-8577, Japan; ^2^NBL Technovator Co., Ltd, Osaka 590-0522, Japan; ^3^Department of Computer Science and Engineering, Shanghai Jiao Tong University, Shanghai 200240, China; ^4^Software College, Northeastern University, Shenyang 110819, China

 Biomedical science and engineering is an interdisciplinary research field, which combines the advanced technologies and problem solving skills with medical and biological science. Since the biomedical solutions generally have large variations and complexity, it is difficult to use a simple way or a classical approach to find the solutions. Computation intelligence techniques such as neural networks and evolutionary algorithms are nature-inspired computational approaches to address complex problems of the real world. Recently, computational intelligence is playing an important role in biomedical research fields, such as computer-aided diagnostics (CAD), computer-aided surgery (CAS), computational anatomy, and bioinformatics. Approaches based on computational intelligence have been shown to be advantageous compared to classical approaches. 

 This special issue focuses on major trend and new techniques in computational intelligence and their use in biomedical science and engineering. We received 15 submissions. Each paper was reviewed by two external referees. We finally accepted 8 papers for our special issue. The area of interest of the accepted papers covers a broad spectrum of computational intelligence techniques with application to biomedical science and engineering. 

 H. Jiang at al. proposed an optimized medical image compression algorithm based on wavelet transform and improved vector quantization, which can maintain the diagnostic-related information of the medical image at a high compression ratio. 

 P. Zhang et al. proposed a composite match index (CMI) method for the integration of different feature-point matching approaches in order to improve the robustness of the matching result. The proposed method has also been applied to interior deformation field measurement of complex human tissues from three-dimensional magnetic resonance (MR) volumetric images.

 C.-L. Lin et al. proposed a hybrid particle swarm optimization (HPSO) for robust medical image registration, which includes two concepts of genetic algorithms—subpopulation and crossover.

 H. Ikeno et al. developed a scheme and tools to construct a standard moth brain for neural network simulations. Morphological models of neurons are reconstructed from confocal image data of neurons.

 Y. Nishitani et al. detected a significantly greater number of Rev. M3 patterns from the time series stimulated spike response than from the random series (interval shuffle) data in neuronal networks formed on MEAs. These results show the possibility of assembling LFSR circuits or its equivalent ones in a neuronal network.

 S. M. Rabiee and H. Baseri developed three different adaptive neurofuzzy inference systems (ANFISs) for estimation of the setting properties of calcium phosphate bone cement. Despite the relatively small amount of data (25 conditions), the proposed method gave satisfying results.

 S. Tamura et al. proposed automutual information-(AM-I) based randomizing method of bin width and location instead of conventional fixed bin setting for analyzing neuron spike trains. In his second paper, they also proposed a model of human society where roles are divided to each person to obtain high performance as a whole, and as a result people play to train their hidden abilities.

 Although the above papers do not make a complete coverage of the computational intelligence in biomedical science and engineering, it provides a flavor of what are the important issues and the benefits of applying computational intelligence to biomedical science and engineering. We would like to thank the authors for submitting their papers to the special issue as well as the reviewers for providing their expertise and making valuable comments. 

